# Functional asymmetry and effective connectivity of the auditory system during speech perception is modulated by the place of articulation of the consonant- A 7T fMRI study

**DOI:** 10.3389/fpsyg.2014.00549

**Published:** 2014-06-11

**Authors:** Karsten Specht, Florian Baumgartner, Jörg Stadler, Kenneth Hugdahl, Stefan Pollmann

**Affiliations:** ^1^Department of Biological and Medical PsychologyUniversity of Bergen, Bergen, Norway; ^2^Department of Medical Engineering, Haukeland University HospitalBergen, Norway; ^3^Department of Experimental Psychology, Otto-von-Guericke UniversityMagdeburg, Germany; ^4^Leibniz Institute for Neurobiology, MagdeburgGermany; ^5^Division of Psychiatry, Haukeland University HospitalBergen, Norway; ^6^Department of Radiology, Haukeland University HospitalBergen, Norway; ^7^NORMENT Senter for Fremragende Forskning OsloNorway; ^8^Center for Behavioral Brain Sciences MagdeburgGermany

**Keywords:** functional magnetic resonance imaging, ultra high field, auditory cortex, place of articulation, VOT, dynamic causal modeling

## Abstract

To differentiate between stop-consonants, the auditory system has to detect subtle place of articulation (PoA) and voice-onset time (VOT) differences between stop-consonants. How this differential processing is represented on the cortical level remains unclear. The present functional magnetic resonance (fMRI) study takes advantage of the superior spatial resolution and high sensitivity of ultra-high-field 7 T MRI. Subjects were attentively listening to consonant–vowel (CV) syllables with an alveolar or bilabial stop-consonant and either a short or long VOT. The results showed an overall bilateral activation pattern in the posterior temporal lobe during the processing of the CV syllables. This was however modulated strongest by PoA such that syllables with an alveolar stop-consonant showed stronger left lateralized activation. In addition, analysis of underlying functional and effective connectivity revealed an inhibitory effect of the left planum temporale (PT) onto the right auditory cortex (AC) during the processing of alveolar CV syllables. Furthermore, the connectivity result indicated also a directed information flow from the right to the left AC, and further to the left PT for all syllables. These results indicate that auditory speech perception relies on an interplay between the left and right ACs, with the left PT as modulator. Furthermore, the degree of functional asymmetry is determined by the acoustic properties of the CV syllables.

## INTRODUCTION

**FIGURE 1:**
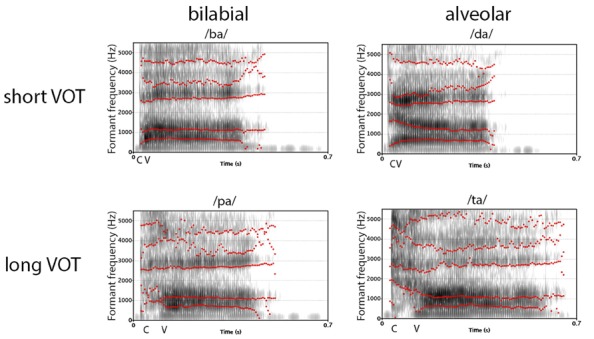
**Comparison of sound spectrogram for the four used CV syllables /ba/, /pa/, /da/.** The contours of the first five formants are displayed, as revealed by the phonetic analysis software Praat (http://www.fon.hum.uva.nl/praat/). The labels “C” and “V” indicate the approximate onset of the consonant and vowel/voice.

A vocal sound is a complex, acoustic event that is characterized by specific spectro-temporal patterns of different speech-sound elements. To perceive a vocal sound as an intelligible speech sound, and particularly to differentiate between consonants, two determining acoustic features are of importance: the “place of articulation” (PoA) and the “voice-onset time” (VOT; Liberman et al., 1958; Lisker and Abramson, 1964b; Voyer and Techentin, 2009). In contrast to vowels that are mainly tonal-sounds with a constant intonation, sounds of stop-consonants are spectrally more complex and are mainly characterized by PoA and VOT. PoA describes the spatial position and configuration of an active articulator that stops the airflow, while VOT describes the time between the release sound of the consonant and the onset of the voice for pronouncing a successive vowel. For example, the consonant–vowel (CV) syllables /da/ and /ta/ have the same PoA but differ in their VOT, as the release sound for /t/ takes longer time than for /d/. Consequently, the onset of the glottal voicing is delayed. On the other hand, /d/ and /t/ share the same configuration of the vocal tract, i.e., they have the same PoA. The PoA of the consonants /d/ and /t/ are called alveolar, with the blockage of the airflow at the alveolar ridge, /b/ and /p/ are called bilabial, since the airflow is stopped at both lips, and, finally, /g/ and /k/ are called velar, with the blockage at the velum. From an acoustic point of view, bilabial stop consonants produce a diffuse spectrum with a primary concentration of energy at 500–1500 Hz, velar stop consonants produce a high-amplitude but low-frequency spectrum with frequencies range from 1500 Hz to 4000 Hz, and, finally, alveolar stop consonants produce, through turbulences at the front teeth, frequencies above 4000 Hz (Cooper et al., 1952; Halle et al., 1957; O’Brien, 1993). Hence, phonological studies often use the stop consonants /b/, /d/, and /g/ that have a short VOT, and /p/, /t/, and /k/ that have a long VOT, and pair them with a vowel, like /a/, /o/, or /i/, to form a CV syllable. In many languages, the syllables /ba/, /da/, and /ga/ are voiced CV syllables with a short VOT, and the CV syllables /pa/, /ta/, and /ka/ are unvoiced syllables with a longer VOT (Liberman et al., 1958; Lisker and Abramson, 1964a; Rimol et al., 2006; Voyer and Techentin, 2009). As can be seen from **Figure 1**, which displays the spectrogram of two associated syllable pairs (ba, pa, and da, ta) that have the same PoA but different VOTs, the spectral pattern differs between the four syllables. In particular, the spectro-temporal pattern over the first 30–70 ms in the frequency range between 0 and 3000 Hz is important for the differentiation of these CV syllables.

Although the differentiation between PoA and VOT is one of the most characteristic elements in the perception of speech, only a few neuroimaging studies, using either functional magnetic resonance (fMRI), electroencephalography, or magnetoencephalography, have tried to address the neuronal underpinnings of VOT and PoA differentiation [see, for example (Gage et al., 2002; Blumstein et al., 2005; Frye et al., 2007; Giraud et al., 2008; Hutchison et al., 2008; Hornickel et al., 2009)]. These studies have shown concordantly that the processing of VOT differences is predominantly performed by the left auditory cortex (AC), while the right AC may have only supporting function but does not linearly follow VOT differences (Frye et al., 2007). Gage et al. (2002) found in their MEG study a longer latency of the M100 component in the right hemisphere for /ba/ but not for /da/ and /ga/. This M100 latency effect is interpreted to reflect a different coding mechanism for syllables with different PoAs, which is also different between the hemispheres. Furthermore, PoA differentiation can already be observed on the level of the brainstem (Hornickel et al., 2009).

CV syllables are quite common in behavioral studies, similar to studies that use a dichotic presentation. Here, two different syllables are presented simultaneously to the left and right ear. Asking the participant to report the syllable they perceived most clearly or earlier, the majority of the population will report the syllable presented to the right ear, irrespective of VOT and PoA. This perceptual bias is called the “right-ear advantage” (REA; Shankweiler and Studdert-Kennedy, 1967; Hugdahl et al., 2009; Westerhausen et al., 2009b). The typical explanation for the REA is the more preponderant neural connection between the right ear and the left AC, while the ipsilateral connection is assumed to be inhibited (Kimura, 1967; Penna et al., 2007). Given that the left hemisphere is the speech dominant hemisphere in the majority of individuals, the REA is taken as a behavioral correlate of the speech lateralization (Hugdahl et al., 1997, 2009). However, taking VOT and PoA into account, it has been shown that different left-right pairings of stop-consonant CV syllables, with different VOTs and/or PoAs to the left and right ear, respectively, can modulate the strength of REA (Speaks and Niccum, 1977; Rimol et al., 2006; Sandmann et al., 2007; Voyer and Techentin, 2009). For example, syllables with long VOTs are generally reported more often, irrespective of the ear to which they have been presented (Rimol et al., 2006). Similarly, Voyer and Techentin (2009) investigated the relationship between PoA and REA, and confirmed an influence of both stimulus dominance as well as VOT on the strength of the REA with velar PoA being the most effective modulator of the REA (Voyer and Techentin, 2009).

These behavioral results could be seen as indication of a bilateral processing of speech sounds, at least on the initial perceptual level, which is also supporting recent models on speech perception (Hickok and Poeppel, 2007; Specht, 2013, 2014). However, these models also confirm the classical view of a predominant function of the left temporal lobe in processing and decoding of speech sounds, but only on a higher hierarchical level than the perception process (Specht, 2013). From animal studies as well as from neuroimaging studies in humans, it is known that the left and right ACs is processing different aspects of an acoustic signal. The left AC is assumed to have a higher spectral and thus higher temporal resolution. The right AC is assumed to integrate the acoustic signal over a longer time window and is expected to be more sensitive to the tonal aspects of a signal (Poeppel, 2003; Boemio et al., 2005). This view has been recently confirmed by electrophysiological results which showed that incoming speech signals are asymmetrically transformed into intrinsic oscillations within the AC. Within the left AC, a higher activity in the gamma frequency band (25–35 Hz) has been observed, while the right AC demonstrated more activity in the theta frequency band (4–8 Hz; Giraud and Poeppel, 2012).

Based on the afore described behavioral and neuroimaging studies, one could hypothesize that CV syllables with different VOTs and PoAs are differently processed by the left and right ACs, due to their different acoustic characteristics. More specific, the higher acoustic complexity of syllables with long VOT are expected to be stronger left lateralized than syllables with a short VOT, since the left AC is expected to have a higher temporal and spectral resolution, while the right AC might be superior in processing the more dominant tonal aspects of a CV syllable with a short VOT. Similarly, differential functional asymmetry has also been reported for PoA (see, e.g., Gage et al., 2002). Based on this, a stronger contribution of the right hemisphere was expected for bilabial than for alveolar syllables. Using a within-speech design, the aim of the present study was to investigate the functional–structural relationship as well as the functional and effective connectivity within the left and right primary and secondary ACs during the perception of CV syllables with different PoAs and VOTs.

To detect these subtle processing differences, this functional magnetic resonance imaging study requires a high sensitivity to subtle differences in the fMRI signal, as well as a reasonable high spatial as well as high temporal resolution, which can be achieved only on an ultra-high-field 7 T MRI.

## MATERIALS AND METHODS

### PARTICIPANTS

Ten healthy, right-handed participants (7/3 male/female, mean age 27, age range 21–39) were investigated in this study. All participants gave written informed consent in accordance with the Declaration of Helsinki and Institutional guidelines. The study was conducted in accordance with the 7 T-MR-protocols approved by the ethics committee of the University of Magdeburg. The participants were recruited from the student population at the University of Magdeburg and the employees at the University hospital Magdeburg, and they got a reimbursement of 30 €. All participants were informed about the rational of the study and they were assured that they could interrupt their participation at any time for any reason. Prior to the scanning, participant got a subject code, containing only two letters and two digits, not related to participant’s name or age. In addition to sound-damping headphones, participants wore earplugs in the scanner.

### STIMULI

In total, eight CV syllables were selected for this study, whereof four syllables started with a bilabial consonant and four with an alveolar consonant. To maximize the contrast between different PoAs, only alveolar and bilabial syllables were used, since they differ the most with respect to their initial frequency distributions (Cooper et al., 1952; Halle et al., 1957; O’Brien, 1993). The used CV syllables were /ba/, /bo/, /da/, /do/, /pa/, /po/, /ta/, and /to/, recorded from four male and four female, non-professional speakers with German as their native language. During the recording of the syllables, speakers read each syllable four times in a randomized order to avoid order effects in the pronunciation. Speakers were standing in front of two AKG 414-B condenser microphones^1^, placed in an echo-reduced chamber. The recordings were made and afterwards edited with Adobe Audition 2.0^2^. Editing of the syllables contained aligning the onsets of the syllables to approximately 20 ms post-onset of the sound file as well as cutting the sound files to a total duration of 700 ms and converting them into a single channel. Using in-house software, written in MATLAB 2011b^3^, the loudness of the sound files was peak normalized to the same level across speakers and syllables.

### DATA ACQUISITION

All MR data were acquired on a 7T Siemens MR system, equipped with an eight-channel head coil (Rapid Biomedical GmbH, Rimpar). Prior to the acquisition of the functional data, a B1 map was obtained, followed by a high-order shim, a T1-weighted Magnetization Prepared Rapid Gradient Echo (MPRAGE) sequence (TE/TR/TI/FA: 5.24 ms/2000 ms/1050 ms/5) and a gradient-echo proton density image (TE/TR/FA: 5.24 ms/1430 ms/5) was acquired. Both datasets were acquired with an isotropic voxel size of 1.0 mm, a matrix size of 256 × 256, and 176 slices. In preparation of the functional images, a separate scan was acquired for estimating the point-spread function needed for the correction of echo-planar imaging (EPI) distortions, typically occurring at ultra-high-field MR systems (Chung et al., 2011). The acquisition of the functional data was separated into five consecutive runs with 220 volumes each. Each volume had 37 interleaved acquired axial slices, an isotropic voxel size of 1.4 mm, a matrix size of 160 × 160, and the EPI sequence had the following parameter: TR = 2000 ms, TE = 23 ms, and FA = 80. To get a suitable temporal resolution for the subsequent dynamic causal modeling analysis, there were no silent gaps between consecutive volumes. The volumes were oriented to cover the AC and the middle and posterior part of the temporal lobe, extending into the inferior part of the parietal lobe, most of the inferior frontal areas, and most of the occipital lobe. An online motion correction was applied during the data acquisition, using the retrospective motion correction, as implemented on MR systems.

Stimulus presentations, data logging, and synchronization with a trigger signal from the MR scanner were all controlled by Presentation software (Version 15.3^4^). Auditory stimuli were presented through MR-compatible stereo headphones (MR confon^5^). A block design was used as experimental design. Within each block, all CV syllables started with the same consonant, but vowels and voices were randomly selected out of the stimulus pool. Each run contained 3 blocks for each PoA and VOT, with 10 trials per block, and the order of the blocks was randomized for each run. Each block of 10 trials lasted 20 s, where the presentation of the syllable lasted for 700 ms, followed by a brief silent period that was randomly assigned to each trial, but restricted in the way that the presentation of these 10 trials together last 20 s. There were an equal number of OFF-blocks, lasting for 16 s, without any presentations, interspersed. During the stimulus presentation phase, participants were asked to listen attentively to the presented syllables. To keep the attention of participants focused onto the syllable presentation but without requesting an active processing of the stimulus content, a few target trials were interspersed where participants were instructed to press a button whenever the sound was presented to the left or right ear only (Specht et al., 2009; Osnes et al., 2011). Therefore, one trial of each block was randomly selected as target trial, where the sound was presented only to one ear. However, these were only 10% of all presented trials, while the other trials were presented bilaterally, where no response was required. In the subsequent analyses, only these non-target trials were considered, where subject attentively listened to the trials but without a motor response. To respond, participants had a response button in their left and right hands. There was no other visual stimulation during the scanning, but subjects were instructed to keep their eyes open.

### DATA ANALYSIS

The fMRI data were processed and statistically analyzed, using SPM8 (SPM = Statistical Parametric Mapping^6^). Prior to the statistical analysis, the data were pre-processed, including the following processing steps: first, a slice-time correction was applied. Although the data were already corrected for head movements and distortions during scanning, possible remaining head movements and head-movement-related image distortions (unwarp) were estimated and corrected using the SPM8 standard procedures but without using an additional phase map. Corrections were made within and across the five runs, using the first image of the first run as reference. Since the images were corrected for geometrical distortions, the fMRI data were co-registered with the anatomical T1 dataset. The latter one was used as source for estimating the stereotactic normalization to a standard reference brain, as defined by a template created by the Montreal Neurological Institute (MNI). This normalization was achieved through, first, a correction of the inhomogeneous distribution of image intensities and thereafter segmentation, using the unified segmentation approach, as implemented in SPM8 (Ashburner and Friston, 2005). Finally, the normalized fMRI data were resampling to a cubic voxel size of 1.5 mm, and a smoothing with a 4 mm Gaussian kernel was applied.

For the statistical analysis, a design matrix was specified that treated the five runs as one single run, by including four additional regressors in the design matrix that accounted for possible baseline shifts between the runs. In preparation of the dynamic causal modeling (DCM) analysis, the data were not modeled as a 2 × 2 design, but rather as an event-related design with two conditions, where the first condition modeled all effects of interest as a parametric design, and the second condition modeled only the target trials, which were not further analyzed, since this task was only included to keep the attention of the participants more constant throughout the study. The first condition was based on the onset of each stimulus, irrespective of PoA or VOT. Without the target trials, these were 540 trials. This main condition is further called the “phonetic input” and represents the general perception of syllables. The additional effects of PoA, VOT, as well as their interaction were added to this first condition as modulating parameters, pooling across voices and vowels. This has the advantage for the subsequent DCM analysis that the system has only one phonetic input, which is the general perception of the syllables, and the PoA and VOT effects can be used as parameter that may modulate connections strength between nodes within the modeled network. The second condition was specified for the target trials, without analyzing them further. This condition was mainly aimed to capture the motor responses, since a response was only required to these target trials. The silent rest periods between stimulations were not explicitly modeled in the design matrix. All onset vectors were convolved with a hemodynamic response function (hrf), as implemented in SPM. During the model estimation, the data were filtered with a high-pass filter with a cut-off of 256 s. Contrasts were estimated for the phonetic input condition as well as for the linear parameter PoA, VOT, and PoA × VOT interaction. Since the silent periods between stimulations were not included in the design matrix as an explicit condition, the contrast for the phonetic input represents the BOLD signal against the implicitly modeled rest condition, while the contrasts for the linear parameter reflect additional activations, on top of the phonetic input. The resulting contrast images were subjected to a second-level group statistics. One sample *t*-tests were performed for the different contrasts. The main effect for the phonetic stimulation was explored with an uncorrected threshold of *p* < 0.001 and a corrected extend threshold of *p*(FWE) < 0.05 per cluster. Localizations of the activations were explored using anatomical templates, included in the MriCron software^7^, as well as using cytoarchitectonic probability maps as implemented in the SPM Anatomy toolbox (Eickhoff et al., 2005).

### DYNAMIC CAUSAL MODELING

Based on the group results gained from the above analyses, five regions of interest were selected. These were the medial and lateral aspects of the AC, bilaterally and, for the left hemisphere, an area covering the planum temporale (PT). Individual time courses were extracted from each region for the most significant voxel from the single-subject analysis that was within a sphere of 8 mm around the peak voxel from the group analysis.

The individual time courses from these five regions were entered into a DCM (version DCM10) analysis. DCM is a generative, statistical approach, applying a neurodynamical model that describes the neuronal activity as non-linear, but deterministic system [see (Friston et al., 2003; Friston, 2009; Penny et al., 2010; Stephan and Roebroeck, 2012) for more details]:

(1) $$\overset{•}{z}=\left(A+\underset{j}{\sum }u_{j}B^{j}\right)z+Cu$$

In this equation, *z* denotes the time course of the neuronal activity and its temporal derivative, respectively, *u* is the experimental input, entering the system at a specified node, while the matrices A, B, and C are defining the model. Thus, three matrices have to be defined: First, the A-matrix represents the functional connection pattern between the nodes, second, the B-matrix parameterized the context-dependent changes in connectivity (effective connectivity), and, finally, the C-matrix defines where the input signal is entering the network. By varying the B-matrix, different DCMs could be specified, forming a model space of different possible solutions, where the most probable solution could be selected by a Bayesian model selection (BMS) approach (Stephan et al., 2009).

Dynamic causal modeling rests on estimating the model evidence that is how good the model explains the data. To find the best model, several models have to be estimated and their model evidences have to be compared (Friston, 2009; Friston et al., 2013). In total, 16 models were specified and a BMS approach (Stephan et al., 2009) was applied for identifying the model with the highest evidence and posterior probability. Common to all 16 models was that the medial and lateral AC of the left and right hemispheres received the phonetic input. Furthermore, a general connection pattern was defined that assumed that an area of the AC is only connected to its neighboring area, to PT, and to its homolog on the other hemisphere, but not to its non-homolog area. For example, the left medial AC was connected to its neighboring left lateral AC, to the planum temporal, and to right medial AC, but not to the right lateral AC. This assumption was confirmed by a pre-analysis on the A-matrix, where fully connected DCM models, i.e., each node was connected to every other node, were compared to DCM models with this reduced connectivity, using a selection approach, based on model families (Penny et al., 2010). Subsequently, the most probable input nodes for these models (C-matrix) were determined in the same way.

The final set of 16 DCM models differed with respect to the modulating influence of PoA on the 16 connections, defined by the A-matrix. Thereby, the B-matrix differed between the 16 models by putting an additional, PoA-dependent weight on the respective connection, while the A- and C-matrices were identical for all models. In general, the strength of a connection is a measure of how activity in one area influences the activity in another area. BMS selection was applied to determine the model with the highest evidence and posterior probability, followed by Bayesian model averaging (BMA). The DCM analysis was restricted to PoA, since the analysis of the activation data revealed significant effects only for PoA (see Section “Results”). However, in an explorative manner, effects of VOT were explored in the same way.

### FUNCTIONAL ASYMMETRY

To examine a possible functional asymmetry, a region of interest (ROI) analysis was conducted based on anatomical probability maps of the AC rather then using manually defined ROIs (Peelle, 2012). To avoid an overlap across ROIs, only the lateral area TE1.2 and the more medial area TE1.1 were considered (Morosan et al., 2001). A mask was created, representing a 50% probability of being either TE1.1 or TE1.2. Individual BOLD signal changes were extracted from these areas and data were subjected to a 2 × 2 × 2 × 2 factorial, repeated-measure ANOVA, with the factor Hemisphere, ROI, PoA, and VOT, and a Greenhouse–Geisser sphericity correction was applied.

For display purposes, data were also extracted for the left PT, based on an anatomical mask.

## RESULTS

**FIGURE 2:**
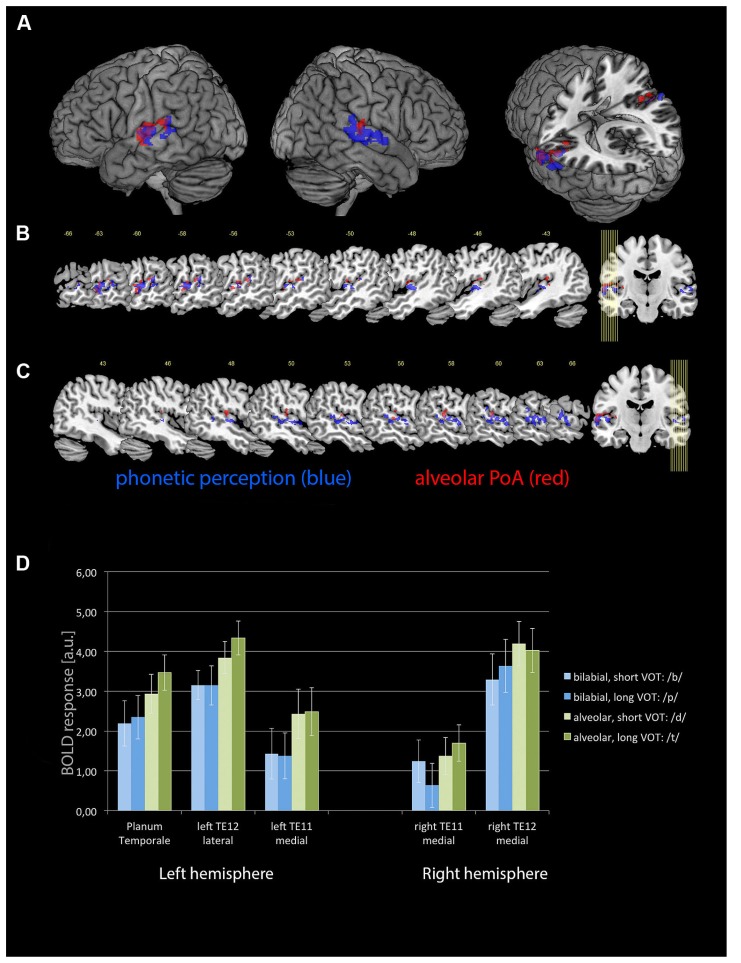
**The activations for the phonetic perception (blue) and specific additional activation for syllables with an alveolar PoA (red) as (A) render views as well as on a selection of sagittal slices for (B) the left hemisphere and (C) the right hemisphere**. A voxel-wise threshold of *p* < 0.001 and a cluster threshold *p*(FWE) < 0.05 was applied to these statistical parametric maps. **(D)** BOLD responses (in arbitrary units) for each of the four different combinations of PoA and VOT, plotted for the medial cytoarchitectonic area TE1.1 and the later area TE1.2 of the left and right ACs (Morosan et al., 2001), as well as for the left PT. Error bars denote standard error.

The phonetic stimulation revealed distinct activations in the left and right ACs, separating into more medial and more lateral aspects of the primary AC and adjacent, secondary areas. Using cytoarchitectonic probability maps (Eickhoff et al., 2005), these areas were identified as TE 1.0, TE 1.1, TE 1.2, and TE 3.0. In addition, there was activation within the left posterior superior temporal gyrus, extending into PT (see **Table 1** and **Figure 2**).

**Table 1:** List of all significant activations for the phonetic perception (across all syllables) and the specific activations for alveolar syllables.

Localization		MNI coordinates			Peak			cluster	
Structure	Cytoarchitectonic	*x*	*y*	*z*	p	*Z*-values	*T*-values	p(FWE-corr)	size (#voxel)
**Phonetic perception**									
Heschl’s gyrus (right)	TE 1.0, TE 1.2, TE 3.0	49.5	-13	1	< 0.001	4.49	9.20	< 0.001	1077
Heschl’s gyrus (left)	TE 1.0, TE 1.2, TE 3.0	-64.5	-11.5	2.5	< 0.001	4.47	9.08	< 0.001	555
Planum temporal (left)	IPC, TE 3.0	-60	-34	14.5	< 0.001	4.30	8.28	< 0.001	228
**Alveolar CV syllables**									
Heschl’s gyrus, superior temporal gyrus (left)	TE 1.2, TE 3.0, STG	-61.5	-8.5	-2	< 0.001	4.74	10.63	< 0.001	432
Heschl’s gyrus (right)	TE 1.0 TE 1.1, IPC/PT	60	-23.5	8.5	< 0.001	4.11	7.43	< 0.001	157
Planum temporal (left)	TE 3, IPC	-64.5	-28	14.5	< 0.001	3.97	6.89	< 0.001	124

*A voxel-wise threshold of *p* < 0.001 and a cluster threshold *p*(FWE) < 0.05 was applied. Activations are described in terms of cluster size [number of voxel (1.5 × 1.5 × 1.5 mm)], peak height, and the respective *p*-values. Furthermore, localizations of effects are described with MNI coordinates, anatomical and cytoarchitectonic structure.*

PoA, VOT, and their interaction were analyzed as parametric modulators, displaying those areas where additional activation to the phonetic stimulation occur. Alveolar CV syllables caused increased activation in the same areas as the phonetic stimulation, but stronger on the left side (see Table 1 and **Figure 2**). In contrast, no additional effects were seen for the bilabial syllables. Furthermore, neither short nor long VOTs caused additional activations at the selected threshold. There was a marginally significant PoA × VOT interaction in medial aspects of the left AC, but only when the extent threshold was reduced to an uncorrected cluster size of *p* < 0.05, while keeping the peak threshold at *p* < 0.001. This interaction indicated that the activation was strongest for syllables with alveolar PoA and long VOT. Based on these results, five regions of interest were identified, with the following MNI coordinates: left lateral AC [-65 -12 3], left medial AC [-48 -15 6], left PT [-60 -34 15], right medial AC [50 -13 1], and right lateral AC [63 -22 7].

### DYNAMIC CAUSAL MODELING

**FIGURE 3:**
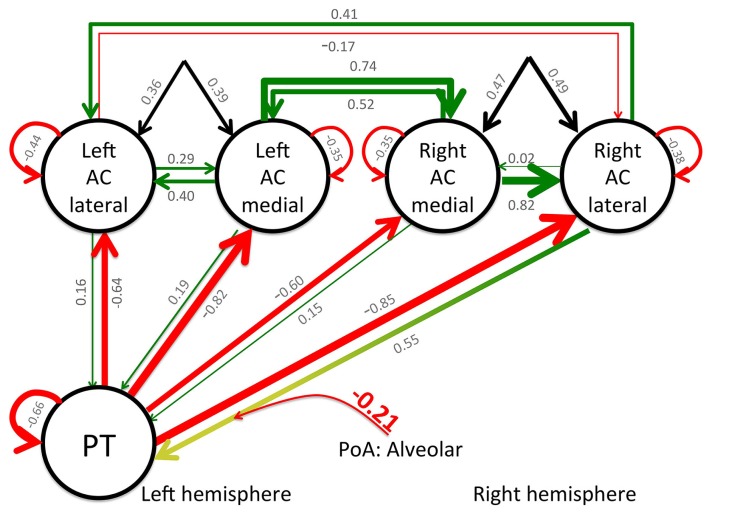
**Results of the DCM analysis: functional and effective connectivity as modeled by the winning model 3**. Bayesian model averaging was applied to estimate the respective connection strength. Green lines indicate a positive connectivity, red lines inhibition, and the black lines indicate the input into the system. The values for the respective connectivity strength are printed in gray for the values from the A- and C-matrices and the red, bold value indicates the value from the B-matrix. To increase readability, the thickness of the lines indicates the respective connectivity strength, as well. Note that the presentation of a CV syllable that starts with an alveolar stop-consonant modulates the effective connectivity between the PT and the right lateral AC.

Dynamic causal modeling analyses were performed for the previously described regions of interest and a BMS analysis was applied to determine the most probable model. The winning model had a relative log-evidence of 280, and a posterior probability (pP) of pP = 1.0, with pP < 0.001 for the remaining 15 models. Finally, BMA was applied to determine the connection strength between the nodes. The overall connection matrix (A-matrix) demonstrated that all feedback connections from PT to the left and right ACs were inhibited when processing CV syllables, irrespective of PoA and VOT. The same pattern was observed for the connection from the left lateral AC to its right homolog. Finally, the B-matrix indicated that CV syllables with alveolar PoA inhibit the forward connectivity from the right lateral AC to PT (see **Figure 3**).

Since an effect for VOT was hypothesized, a corresponding DCM analysis was performed for VOT, although there was no significant effect for VOT in the voxel-wise analyses. In accordance with that, BMS did not favor a single model. However, two models had relatively high posterior probabilities (*pP* > 0.4). Both models had in common that the connection from the left lateral (model 1) or medial (model 2) AC to the respective homolog was increased. However, since neither the voxel-wise analysis nor the DCM analysis gave evidence for a systematic VOT effect, these results were not followed up.

### FUNCTIONAL ASYMMETRY

The 2 × 2 × 2 × 2 repeated-measure ANOVA with the factor hemisphere, ROI, PoA, and VOT revealed significant main effects of PoA [*F*(1,9) = 48.007, *p* < 0.001] and ROI [*F*(1,9) = 33.813, *p* < 0.001], while the main effects for hemisphere and VOT became not significant (both *p* > 0.3). In addition, there was a significant PoA × hemisphere interaction [*F*(1,9) = 10.049, *p* < 0.011]. There were no other significant interaction effects. *Post hoc* tests, using Fisher LSD test, revealed that there was higher activity for alveolar syllables in the left than right hemisphere (*p* < 0.0005), and that alveolar syllables caused in both hemispheres higher activity than bilabial syllables (left hemisphere *p* < 0.0001, right hemisphere *p* < 0.0001). In contrast, the activity caused by bilabial syllables was not different between the left and right hemispheres (*p* = 0.4; see **Figure 2D**).

## DISCUSSION

Using a within-speech design, where only CV syllables with different PoAs and VOTs were contrasted, the present study aimed to investigate the differential functional-asymmetry as well as modulation of the functional and effective connectivity as a function of PoA and VOT. To achieve this goal, this study depended on the superior sensitivity and high spatial resolution of a high-field 7 T MR scanner.

The results demonstrate that phonetic perception is a highly complex process that rests on the interaction and complementary processing properties of the left and right primary and secondary ACs. The overall phonetic perception revealed the expected pattern of bilateral activations within the left and right posterior temporal lobe, centred in the respective primary ACs. On this general level of phonetic perception, no strong functional asymmetry could be observed, as confirmed by the ROI analysis where no main effect of hemisphere was detected. However, syllables with an alveolar PoA increased the activation particularly in the left hemisphere, comprising the AC and extending posteriorly into PT. This was further confirmed by the ROI analysis, demonstrating the highest activation within TE1.1 and TE 1.2 of the left hemisphere for alveolar CV syllables. This result was paralleled with a decreased connectivity between the lateral right AC and the left PT. By contrast, neither short nor long VOT alone resulted in increased activations at the selected threshold, but an interaction of PoA and VOT was observed at a lower cluster extend threshold.

In general, these results indicate a context-dependent degree of lateralization on the level of the primary AC. This is further supported by three important results from the DCM analysis. First, all feedback connections from PT to the medial and lateral parts of the primary AC of the left and right hemisphere are inhibited during the perception of speech sounds, irrespective of PoA and VOT. Furthermore, the winning model indicated that the forward connection from the right lateral AC to PT is inhibited during the processing of alveolar syllables, increasing the inhibitory influence of PT on the right lateral AC. Although the connectivity from the right medial AC to PT remained unchanged, the inhibition of the connection between the right lateral AC and PT may indicate that activity in PT has a suppressing influence on the activity in the right lateral AC. Supporting evidence for this interpretation comes from the ROI analysis, demonstrating only marginal activation differences between bilabial and alveolar CV syllables (see **Figure 2D**). In contrast, increased activity has been observed within the left AC and PT during the perception of alveolar CV syllables. Finally, the back connection from the left to the right lateral primary AC is generally inhibited during the processing of these syllables, which indicates a directed information flow from the right to the left lateral primary AC. Supporting evidence for this view comes from dichotic listening studies. Correlating diffusion tensor imaging data with dichotic listening performance, the connection strength between the left and right ACs determines the strength of the right ear advantage. A higher structural connectivity between the posterior superior temporal areas of both hemispheres cause a higher rate of correct left ear responses (Westerhausen and Hugdahl, 2008; Westerhausen et al., 2009a). The auditory commissures cross in the splenial region of the corpus callosum (Westerhausen et al., 2009a). Splenial lesions diminish the number of correct left ear responses in dichotic listening (Pollmann et al., 2002).

The presented DCM results support the view of a superior processing capacity of the left AC for spectrally complex sounds as opposed to the right AC that is assumed to process more tonal sounds, like vowels. However, the results also indicate a more differentiated view on this asymmetry, since the activation results, the ROI analysis, as well as DCM results demonstrate a functional asymmetry toward the left, particularly for the processing of syllables with an alveolar consonant. This speaks against a simple dichotomising of a left lateralized processing of consonants and a more right lateralized processing of vowels (cf. Rimol et al., 2006). The results rather suggest a constant and context depended variation of the functional asymmetry during speech perception, supporting the notion that the inherent acoustic complexity of speech sounds has to be taken more into account in studies of functional asymmetry (McGettigan and Scott, 2012). Moreover, the perception and decoding of speech signals has to be seen as an integrated interplay of the left and right auditory system across the theoretical concepts of consonants and vowels. In the present study, however, the analysis was averaged across vowels. Therefore, differential effects through co-articulation, that is the influence of the subsequent vowel on the articulation of the consonant, are eliminated, but future studies may focus on differential effects of co-articulation, as well.

Both the activation data and the DCM results support the known importance of PT in processing complex auditory information, such as speech signals (Binder et al., 1996; Griffiths and Warren, 2002; Price, 2012; Specht, 2014), although PT contribution has also been associated to auditory processing of non-verbal stimuli, spatial hearing, as well as auditory imagery (Binder et al., 1996; Specht and Reul, 2003; Specht et al., 2005; Obleser et al., 2008; Isenberg et al., 2012; Price, 2012). PT is an anatomical structure at the posterior end of the superior temporal gyrus, at the intersection with the parietal lobe. PT is typically larger on the left than on the right hemisphere (Binder et al., 1996; Preis et al., 1999; Dorsaint-Pierre et al., 2006), which was originally taken as evidence for a prominent role in speech perception- a view that has been revised toward a more general role in auditory processing (Griffiths and Warren, 2002; Krumbholz et al., 2005). Interestingly, intracranial recordings revealed categorical speech processing in the posterior superior temporal gyrus, including PT, when presenting a stimulus continuum that gradually varied PoA by changing between the voiced CV syllables /ba/ - /da/ - /ga/ (Chang et al., 2010). Concordantly, the current results confirm that PT could be seen as a computational hub for complex auditory processing that has not only a high sensitivity to speech sounds, but also presents a differential response and connectivity pattern to CV syllables with different PoA. The observed inhibitory effect of PT onto the right AC may therefore be a general process, not restricted to the processing of verbal sounds, but spectrally complex sounds *per se*.

The distinct contribution of lateral and medial aspects of the AC may reflect the different subfields of the AC, forming auditory field maps with orthogonal tonotopic and periodotopic gradients (Barton et al., 2012). Given the high spatial resolution and the high functional sensitivity of the applied method, the observed differential contribution of lateral and medial parts of the AC together with dominating right-to-left connectivity of the lateral AC, and higher medial-to-lateral connectivity within the right AC, may reflect the different contribution of these auditory field maps to the speech perception process (see **Figure 3**). Interestingly, there are no observed changes of any connectivity for the left AC, which possibly reflects its constant involvement in the processing of speech stimuli, while the right AC demonstrates a more context-dependent contribution.

In contrast to our *a priori* hypothesis, the present study could not detect a generally increased leftward asymmetry for syllables with a long VOT, irrespective of PoA. However, it should be emphasized that a trend toward a leftward asymmetry for long VOT syllables were observed at more liberal thresholds.

Finally, there are also limitations in the present study. First of all, ultra-high-field fMRI substantially increases the spatial resolution of fMRI and increases the temporal resolution to a certain extent, as well. However, these benefits are compromised by non-homogenous distribution of voxel values (Watanabe, 2012) and substantial susceptibility artifacts through ghosting and movement (Beisteiner et al., 2011), as well as image distortions, which have to be corrected (Chung et al., 2011). Often affected areas with reduced sensitivity to the BOLD signal are, for example, inferior parts of the temporal lobe. One has to bear in mind that possible activations in those areas may not have reached significance.

Second, one has to be cautious in generalizing these results to all languages, since different languages stress stop-consonants differently. However, it is still reasonable to assume that PoA and/or VOT act as modulator of functional asymmetry in most languages.

In summary, all results are broadly in line with the notion that the left and right ACs demonstrate a division of labor in processing speech sounds. More specifically, we observed a varying pattern of functional left–right asymmetry that depended on the spectral complexity of the consonant sounds. Alveolar syllables generally caused a stronger activation of the left AC, including PT, as well as a reduced connectivity from the right lateral AC to the left PT, indicating a possible inhibitory effect of PT on the right AC during processing of spectrally more complex CV syllables.

## Conflict of Interest Statement

The authors declare that the research was conducted in the absence of any commercial or financial relationships that could be construed as a potential conflict of interest.

## Footnotes

1 www.akg.com

2 www.adobe.com

3 www.mathworks.com

4 http://www.neurobs.com

5 http://www.mr-confon.de

6 www.fil.ion.ucl.ac.uk/spm

7 http://www.mccauslandcenter.sc.edu/mricro/mricron/
